# Role of tissue transglutaminase in the pathogenesis of diabetic cardiomyopathy and the intervention effect of rutin

**DOI:** 10.3892/etm.2015.2223

**Published:** 2015-01-28

**Authors:** HAI-CHENG GAO, KUN ZHU, HAI-MEI GAO, CHUN-SHENG MIAO, LE-NING ZHANG, WEI LIU, HUA XIN

**Affiliations:** 1 Department of Clinical Pharmacy and Pharmaceutical Management, Jilin University, Changchun, Jilin 130021, P.R. China; 2 Department of Endocrinology, The 208th Hospital of PLA, Changchun, Jilin 130000, P.R. China; 3 Patient’s Information Recording Room, The Second Hospital of Jilin University, Changchun, Jilin 130041, P.R. China; 4 Department of Chest Surgery, The China-Japan Union Hospital of Jilin University, Changchun, Jilin 130000, P.R. China

**Keywords:** diabetic, diabetic cardiomyopathy, tissue transglutaminase, rutin

## Abstract

The aim of this study was to investigate the role of tissue transglutaminase (tTG) in the pathogenesis of diabetic cardiomyopathy (DCM) and the intervention effect of rutin. DCM was induced in rats by the injection of streptozotocin (STZ; 25 mg/kg). After a preliminary examination, the rats were randomly divided into four groups: Control (n=8), STZ-induced DCM (n=8), STZ + positive drug (captopril; n=6) and STZ + rutin (n=8) groups. The DCM model was evaluated using blood sugar values, serum enzyme levels, hematoxylin and eosin staining and Masson’s staining, *ex vivo*. The protein and mRNA expression of tTG was assessed with immunohistochemistry, western blotting and reverse transcription-quantitative polymerase chain reaction (RT-qPCR). The rat model of DCM was successfully established by STZ administration, and the expression levels of tTG were significantly increased in the DCM model. Following the injection of captopril or rutin, the blood sugar values, collagen content and expression levels of tTG were gradually reduced and serum enzyme levels were increased, as compared with those in the STZ-induced DCM group. In conclusion, tTG plays an important role in STZ-induced DCM. In addition, rutin may inhibit the expression of tTG and regulate myocardial injury in STZ-induced DCM.

## Introduction

Diabetes mellitus (DM) can cause many complications, including heart disease, kidney failure (1) and diabetic cardiomyopathy (DCM) (2). DCM can lead to the heart being unable to circulate blood through the body effectively (heart failure), with accumulation of fluid in the lungs or legs (3). At present, heart failure in people with diabetes primarily results from coronary artery disease, and DCM is only considered to exist if no coronary artery disease is present to explain the heart muscle disorder (4). Numerous factors have been identified to be involved in the pathogenesis of DM and cardiomyopathy, including MMP-2 and ICAM-1 (5).

Tissue transglutaminase (tTG), a member of the transglutaminase family (6), is Ca^2+^ dependent (7) and is reported to play an important role in myocardial injury (8). tTG is present in the intracellular and extracellular spaces of various types of tissues and in various organs, including the heart and liver (8). In the extracellular space, tTG is associated with cell adhesion, extracellular matrix stabilization, wound healing, receptor signaling, cellular proliferation and cellular motility (9). It is unclear whether tTG plays an important role in STZ-induced DCM, and if it does, whether drugs can be used to inhibit the expression of tTG and regulate the occurrence and development of DCM. In the past decade, numerous drugs with potential for the treatment of DCM have been identified, for example sulforaphane (10), but the treatment effect requires improvement. Therefore, the development of drugs to treat DCM is urgently necessary. Rutin has the apparent ability to strengthen blood vessels, and can reduce and delay the development of diabetes (11). However, whether rutin is able to prevent and treat DCM is unclear.

The present study investigated blood sugar values, cardiac enzyme levels, the expression of tTG and collagen I (Col-I) in the heart and the interventional effect of rutin in a rat model of DCM.

## Materials and methods

### Materials

Streptomycin avidin peroxidase immunohistochemistry (SP-IHC) kits for tTG and Col-I were purchased from Zhongshan Jinqiao Biotechnology Co., Ltd. (Beijing, China). Captopril tablets were from Zhengzhou Ruikang Pharmaceutical Co., Ltd. (Zhengzhou, China), rutin was from Xi’an Tianxingjian Industry Co., Ltd. (Xi’an, China) and streptozotocin (STZ) was from Sigma-Aldrich (St. Louis, MO, USA). All other chemicals were obtained from commercial suppliers in China.

### Animals

A total of 30 male Wistar rats (SCXKJ2007-0003; 180–220 g) were purchased from Jilin University Laboratory Animal Center (Changchun, China) and were randomized into four groups: control (n=8), STZ-induced DCM (n=8), STZ + positive drug (captopril tablets; n=6) and STZ + rutin (n=8) groups. The rat model of DCM was established by the peritoneal injection of STZ (25 mg/kg). Captopril and rutin were administered at doses of 5 and 40 mg/kg/day, respectively, by gastric lavage. The treatment began 21 days after the injection of STZ and was administered for 28 days. At the end of the experiments (day 49 after STZ injection), the rats were sacrificed and their hearts were removed. A portion of the heart was fixed in 10% phosphate-buffered formalin, frozen in liquid nitrogen and stored at −80°C for extraction. The study was approved by the ethics committee of the College of Pharmacy of Jilin University (Changchun, China).

### Blood sugar values

The Wistar rats were reared for one month by feeding with a high-sugar diet. The blood sugar levels of the rats were then measured using test strips (Sannuo Bio-Sensing Technology Co., Ltd., Changsha, China).

### Analysis of serum enzymes

Serum aspartate aminotransferase (AST), lactic dehydrogenase (LDH), creatine kinase (CK) and creatine kinase isoenzyme MB (CK-MB) activities were measured using the MD-100 Multifunctional Automatic Biochemistry Analyzer (Sanhe Medical Equipment Co., Ltd., Dandong, China) according to the manufacturer’s instructions.

### Hematoxylin and eosin (H&E) and Masson’s staining

Renal histology was assessed by light microscopy with H&E staining and Masson’s trichrome staining. Heart tissues were harvested and fixed in neutral buffered 10% or 4% paraformaldehyde. After paraffin embedding, heart tissue sections were prepared (0.5 μm) and then subjected to H&E and Masson’s staining for morphological observation using a PM-10AO automatic photomicrographic device (Olympus Co., Ltd., Tokyo, China). Ten high-power microscopic fields were randomly selected and the percentage of Masson staining was determined from the average gray value of five rats in each group.

### Immunohistochemical staining

The paraffin-embedded tissue sections (0.5 μm) were deparaffinized with xylene and rehydrated with graded washes of ethanol to phosphate-buffered saline (PBS). Staining was conducted using SP-IHC kits. In this process, each slice was treated with 30 μl 3% H_2_O_2_ (reagent A), incubated at room temperature for 20 min and washed twice with PBS. Then, 30 μl goat serum (reagent B) was added, followed by incubation at room temperature for 20 min and two washes with PBS. Each slice was incubated in 30 μl primary antibody and placed in a wet box at 4°C overnight. The primary antibody was rabbit anti-rat tTG polyclonal antibody (1:200 dilution) or rabbit anti-rat Col-I monoclonal antibody, (1:150 dilution). After washing with PBS, the slices were incubated in 30 μl biotinylated polyclonal secondary antibody (reagent C) at room temperature for 30 min, followed by washing with PBS. The diaminobenzidine (DAB) method was used for color development, followed by washing with tap water. Slices were restained with hematoxylin, incubated in ammonia, dehydrated with gradient ethanol, transparentized with xylene and finally sealed with neutral gum. The cells with brown particles in their cytoplasm and nucleus when observed under a light microscope were considered positive. The expression levels were determined as the average gray value of five rats in each group.

### Western blotting

Heart tissues were homogenized in protein lysis buffer and 50 g proteins were separated on 10% sodium dodecyl sulfate (SDS) gels and electroblotted to polyvinylidine fluoride membranes. The membranes were blocked with skimmed milk powder solution for 2 h. Blots were incubated using rabbit anti-rat tTG polyclonal antibody (Santa Cruz Biotechnology, Inc., Dallas, TX, USA) at 4°C overnight, followed by peroxidase-conjugated goat anti-rabbit IgG polyclonal antibody (dilution 1:2,000: Boster Bio-Engineering Co., Ltd, Wuhan, China) for 2 h at 37°C. Color was developed with an enhanced chemiluminescence (ECL) reagent (Thermo Fisher Scientific Inc., Waltham, MA, USA) in a dark room. The gray scale values were analyzed using the Tanon-1000 Gel Image System (Tanon, Shanghai, China).

### Reverse transcription-quantitative polymerase chain reaction (RT-qPCR)

Heart tissue (100 mg) was ultrasonically homogenized with 1 ml TRIzol, transferred into Eppendorf tubes and incubated at room temperature with 0.2 ml chloroform for 5 min. The samples were then shocked at room temperature (15–30°C) for 15 sec, incubated for 2–3 min and centrifuged at 12,000 × g for 15 min (2–8°C). The supernatant was transferred into a new tube. To each tube was added 0.5 ml isopropanol at room temperature, and after 10 min the tubes were centrifuged at 12,000 × g for 10 min. The supernatant was removed, 1 ml 75% ethanol was added and the mixture was centrifuged at 7,500 × g for 5 min to provide total RNA. A sample of the total RNA (2 μl) was reversed transcribed to cDNA. In this process, the RNA was dissolved in 30 μl diethylpyrocarbonate-treated water. First-strand DNA was synthesized using an RT reaction system (20 μl) as follows: 10 μl deionized water with no RNA enzyme, 2 μl template RNA, 180 μl Oligo(dT), 4 μl 5X reaction buffer, 1 μl RNase inhibitor (20 U/μl), 2 μl dNTP mix (10 mmol/l) and 1 μl SuperScript II RT (TransGen Biotech Co., Ltd., Beijing, China). The reaction conditions were as follows: 70°C for 5 min, 37°C for 5 min, 37°C for 60 min, 70°C for 10 min and then placed on ice for subsequent testing or preservation at −80°C. The primers for tTG were 5′-AAGGGAAGTCTTCACCAGAGCCAA-3′ (sense) and 5′-CGATGTGGGCAAACACGTCAAAGT-3′ (anti-sense). PCR was conducted using Taq DNA polymerase (TransGen Biotech Co., Ltd.) and the following amplification protocol: 1 cycle at 95°C for 5 min followed by 30 cycles consisting of 30 sec at 95°C, 45 sec at 55°C and 1 min at 72°C, and a final 10 min extension at 72°C in an automated thermal cycler (PCR Express; Thermo Fisher Scientific).

### Statistical analyses

All statistical analyses were performed using SPSS version 15.0 for Windows (SPSS Inc., Chicago, IL, USA). Comparisons between multiple groups were performed by one-way analysis of variance (ANOVA). P<0.05 was considered to indicate a statistically significant difference.

## Results

### Blood sugar values

Blood sugar measurements were obtained from the 30 rats. As shown in Table I, the blood sugar levels in the model group were higher than those in the control group (P<0.05). This indicated that the diabetic rat model was successfully constructed. The blood sugar levels in the STZ + positive drug and STZ + rutin groups were lower than those in the model group on days 42 and 49 (P<0.05). These results indicate that rutin may have a role in blood sugar reduction.

### Serum enzyme values

The activity levels of AST, LDH, CK and CK-MB in the model group were lower than those in the control group (P<0.05). These results showed that STZ might induce myocardial injury. The activity levels of AST, LDH, CK and CK-MB in the STZ + positive drug and STZ + rutin groups were higher than those in the model group (P<0.05). These results indicate that rutin may have a role in increasing the activity levels of AST, LDH, CK and CK-MB (Table II).

### H&E staining

**Figure 1:**
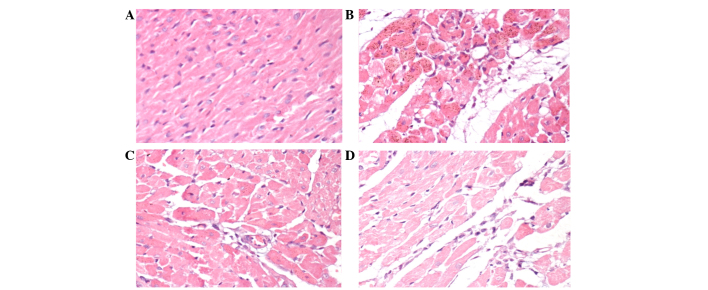
Histopathological images. Tissue samples were preserved in 10% neutral buffered formalin, paraffin embedded, processed to slides, and stained with hematoxylin and eosin. Representative histopathological images are shown. (A) Normal, (B) model (STZ-induced DCM), (C) STZ + positive drug (captopril) and (D) STZ + rutin. Magnification, ×400. STZ, streptozotocin; DCM, diabetic cardiomyopathy.

A morphological assessment, as the gold standard for disease diagnosis, is the most reliable method for diagnosing organ injury. Examination of the rat heart tissue slices revealed that there was evident denaturation and edema in the apex cordis and endocardium following the injection of STZ. Myocardial tissues appeared necrotic in the endocardium and interstitium. These results suggest that heart injury was successfully generated. Following the injection of captopril or rutin, there was a certain improvement in the degree of myocardial injury. These results indicated that rutin may have a protective effect against STZ-induced myocardial injury (Fig. 1).

### Masson’s staining

**Figure 2:**
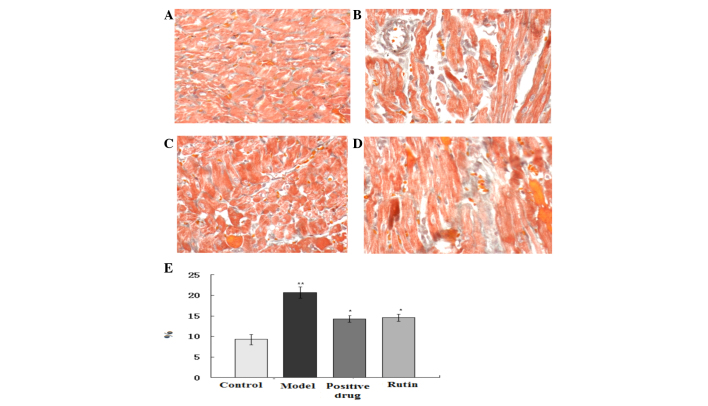
Masson trichrome staining. (A) Normal, (B) model (STZ-induced DCM), (C) positive drug (STZ + captopril) and (D) rutin (STZ + rutin). (E) Percentage of Masson trichrome staining in the various groups. STZ, streptozotocin; DCM, diabetic cardiomyopathy. Magnification, ×1,000. ^*^P<0.05 vs. model; ^**^P<0.01 vs. control.

Experimental results demonstrated that the collagen content in the model group was higher than that in the control group following the injection of STZ (P<0.05). The collagen contents in the groups treated with captopril or rutin were lower than that in the model group (P<0.05). These results indicate that treatment with rutin reduces collagen content (Fig. 2).

### Col-I staining

**Figure 3:**
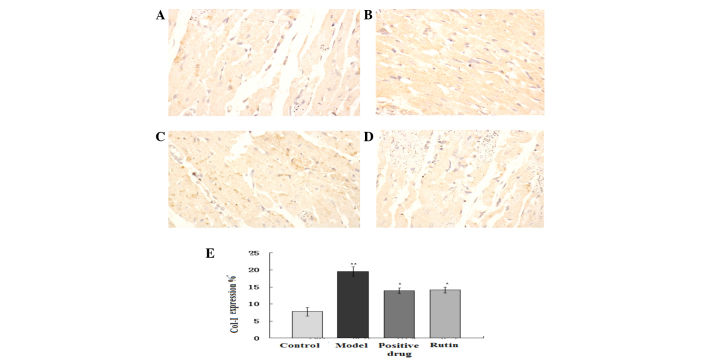
Immunohistochemistry of collagen I (Col-I). (A) Normal, (B) model (STZ-induced DCM), (C) positive drug (STZ + captopril) and (D) rutin (STZ + rutin). (E) Percentage of Col-I staining in the various groups. STZ, streptozotocin; DCM, diabetic cardiomyopathy. Magnification, ×1,000. ^*^P<0.05 vs. model; ^**^P<0.01 vs. control.

As shown in Fig. 3, the expression level of Col-I in the model group was higher than that in the control group (P<0.05). After treatment with captopril or rutin, the expression level of Col-I was lower than that in the model group (P<0.05).

### tTG staining

**Figure 4:**
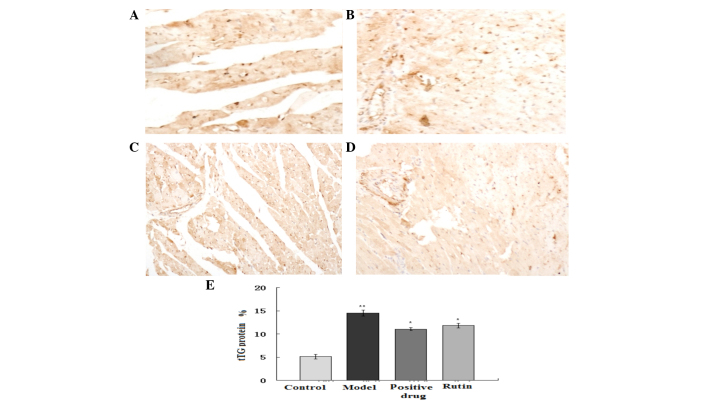
Tissue transglutaminase (tTG) immunohistochemical staining. (A) Normal, (B) model (STZ-induced DCM), (C) positive drug (STZ + captopril) and (D) rutin (STZ + rutin). (E) Percentage of tTG staining in the various groups. STZ, streptozotocin; DCM, diabetic cardiomyopathy. Magnification, ×1,000. ^*^P<0.05 vs. model; ^**^P<0.01 vs. control.

In the control group, tTG immunoreactivity was weakly detected in the myocardial interstitial region (Fig. 4A). In this group, tTG immunoreactivity was observed in blood vessel-like structures. The expression level of tTG increased markedly in the model group as compared with that in the control group (P<0.05). The expression levels of tTG in the groups treated with captopril or rutin were observed to be lower than that in the model group (P<0.05). These results suggest that treatment with rutin inhibits the expression of tTG.

### Expression of tTG as determined by western blotting

**Figure 5:**
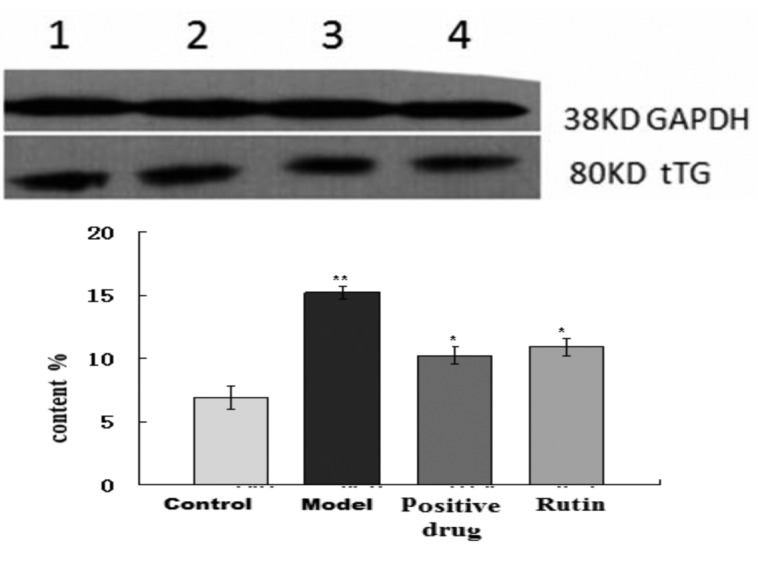
Results of western blotting. Representative western blots and tTG expression levels in the various groups. Lane 1, normal (control) group; lane 2, model (STZ-induced DCM); lane 3, positive drug (STZ + captopril); lane 4, rutin (STZ + rutin). STZ, streptozotocin; DCM, diabetic cardiomyopathy; tTG, tissue transglutaminase. ^*^P<0.05 vs. model; ^**^P<0.01 vs. control.

Western blotting was used to investigate the expression of tTG. The results were analyzed semi-quantitatively according to the grayscale value. As shown in Fig. 5, the expression level of tTG in the model group was higher than that in the model group (P<0.05). The expression levels of tTG protein in the rats treated with captopril or rutin were lower than that in the model group (P<0.05).

### RT-qPCR results

**Figure 6:**
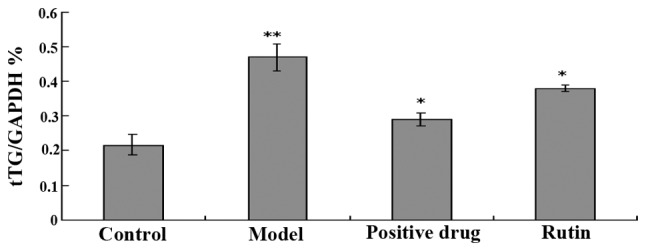
Results of RT-qPCR. mRNA expression of tTg in the rat myocardium of the control, model (STZ-induced DCM), positive drug (STZ + captopril) and rutin (STZ + rutin) groups. RT-qPCR, reverse transcription-quantitative polymerase chain reaction. tTG, tissue transglutaminase; STZ, streptozotocin; DCM, diabetic cardiomyopathy. ^*^P<0.05 vs. model; ^**^P<0.01, vs. control.

RT-qPCR was used to investigate the mRNA expression of tTG. As shown in Fig. 6, the mRNA expression level of tTG in the model group was higher than that in the control group (P<0.05). The expression levels of tTG mRNA in the rats treated with captopril or rutin were lower than that in the model group (P<0.05).

## Discussion

In patients with DM, the cardiac structure and function may be altered in the absence of changes in blood pressure and coronary artery disease, a condition known as DCM. DCM is ventricular dysfunction that occurs independently of coronary artery disease and hypertension. In DCM, the diastolic dysfunction becomes more apparent in the presence of hypertension or myocardial ischemia (12). tTG is the autoantigen in coeliac disease, an illness in which dietary gluten causes a pathological immune response resulting in inflammation of the small intestine and subsequent villous atrophy (13). In the present study, the changes of tTG in the myocardial interstitial region were observed following STZ-induced myocardial injury. The results indicated that the expression levels of tTG protein and mRNA were increased in the myocardial interstitial region following injury. A previous study reported that the expression levels of tTG protein and mRNA in normal human organs were lower than those in injured organs (14). Furthermore, other researchers have suggested a role for tTG in heart mechanisms (15). In a previous study, significant increases of tTG protein and mRNA transcription levels were observed in ischemic Wistar rats at five months following STZ-induction compared with the levels in the sham-injured heart (16). These results indicate that myocardial injury is induced by the injection of STZ. Rutin has strong antioxidant properties, and the ability to chelate metal ions, such as iron, and reduce the Fenton reaction (13). Rutin is important due to its effect in strengthening capillaries. A previous animal study showed that it has preventive and healing effects (17). This indicates the possibility that rutin may play an important role in DCM. In the present study, the expression levels of tTG protein and mRNA, blood sugar levels, serum enzyme levels and collagen content were reduced gradually in the DCM model rats following the injection of rutin. These results indicate that rutin may have a preventive effect for DCM.

In conclusion, tTG may play an important role in STZ-induced DCM. In addition, rutin may inhibit the expression of tTG protein and gene, and regulate blood sugar values and serum enzymes levels. However, the results require verification in further studies.

## Figures and Tables

**Table I:** Blood sugar levels in each group (mmol/l).

Groups	N	35 days	42 days	49 days
Control	8	6.20±0.55	5.50±0.24	6.44±0.24
STZ-induced DCM	8	18.10±2.41^a^	18.00±2.81^a^	16.50±3.50^a^
STZ + positive drug	6	15.01±6.80^b^	13.90±3.18^b^	14.20±2.63^b^
STZ + rutin	8	18.36±2.46	14.21±2.63^b^	15.85±3.05

Values are mean ± standard deviation. STZ, streptozotocin; DCM, diabetic cardiomyopathy; positive drug, captopril.

a P<0.05 vs. control,

b P<0.05 vs. STZ-induced DCM.

**Table II:** Serum enzyme values in each group (μ/l).

Groups	N	AST	LDH	CK	CK-MB
Control	5	100.6±6.7	1834.4±429.9	1002.2±120.0	999.9±128.0
STZ-induced DCM	5	124.8±17.4^a^	2255.4±69.1^a^	1341.6±124.9^a^	1339.3±120.3^a^
STZ + positive drug	5	102.8±2.0^b^	1999.4±135.6^b^	1198.6±65.3^b^	1023.5±90.8^b^
STZ + rutin	5	113.0±4.4^b^	2199.2±93.7	1419.0±120.5	1333.8±112.9

Values are mean ± standard deviation. STZ, streptozotocin; DCM, diabetic cardiomyopathy; positive drug, captopril; AST, aspartate aminotransferase; LDH, lactate dehydrogenase; CK, creatine kinase; CK-MB, creatine kinase isenozyme MB.

a P<0.05 vs. control,

b P<0.05 vs. STZ-induced DCM.
